# Prey Capture Behavior in an Arboreal African Ponerine Ant

**DOI:** 10.1371/journal.pone.0019837

**Published:** 2011-05-11

**Authors:** Alain Dejean

**Affiliations:** 1 Centre National de la Recherche Scientifique (CNRS), Écologie des Forêts de Guyane (UMR-CNRS 8172), Campus Agronomique, Kourou, France; 2 Université de Toulouse (Université Paul Sabatier), Toulouse, France; University of Arizona, United States of America

## Abstract

I studied the predatory behavior of *Platythyrea conradti*, an arboreal ponerine ant, whereas most species in this subfamily are ground-dwelling. The workers, which hunt solitarily only around dusk, are able to capture a wide range of prey, including termites and agile, nocturnal insects as well as diurnal insects that are inactive at that moment of the Nyctemeron, resting on tree branches or under leaves. Prey are captured very rapidly, and the antennal palpation used by ground-dwelling ponerine species is reduced to a simple contact; stinging occurs immediately thereafter. The venom has an instant, violent effect as even large prey (up to 30 times the weight of a worker) never struggled after being stung. Only small prey are not stung. Workers retrieve their prey, even large items, singly. To capture termite workers and soldiers defending their nest entrances, ant workers crouch and fold their antennae backward. In their role as guards, the termites face the crouching ants and end up by rolling onto their backs, their legs batting the air. This is likely due to volatile secretions produced by the ants' mandibular gland. The same behavior is used against competing ants, including territorially-dominant arboreal species that retreat further and further away, so that the *P. conradti* finally drive them from large, sugary food sources.

## Introduction

Despite extreme climatic conditions and the unpredictable availability of prey, ants dominate the invertebrate communities in tropical rainforest canopies where they often represent ca. 50% of the animal biomass and 90% of the individuals [1]–[3]. Yet, on a geological time-scale, ants have not always been so present in arboreal environments. They likely began to become arboreal with the arrival of angiosperms, something that has also driven the diversification of major herbivorous insects, including hemipterans [4]–[6].

This very high abundance is possible because most ant species are at least partially herbivorous, feeding on extrafloral nectar (EFN), food bodies (FBs), pollen, fungal spores and mycelium, epiphylls, and sap. They are even considered “cryptic herbivores” when they attend sap-sucking hemipterans for their honeydew [7]–[10]. Energy-rich EFN, FBs and Hemipteran honeydew fuel their efforts in predation and the defense of their territory and host plant [11], [12]. This numerical abundance is mostly represented by “territorially-dominant” arboreal ants whose very populous colonies defend absolute territories (several neighboring trees) that are distributed in a mosaic pattern, creating what has become known as “arboreal ant mosaics”. These ants tolerate the presence of “non-dominant” species with small colonies on their territories [11], [13]–[15].

Since most prey in the tree foliage are insects able to escape by flying away, jumping or dropping, arboreal ants have optimized their ability to capture such insects in this restricted foraging area. In the territorially-dominant arboreal ant species and plant-ants studied so far, workers ambush in a group permitting them to capture a wide range of insects that are spread-eagled, and only certain species need to use their venom [11], [16]–[24]. On the contrary, the workers of non-dominant species forage solitary and their success depends on the rapidity of their attack and very effective venom, something noted in two ponerine species: *Pachycondyla goeldii* and *Platythyrea modesta* [25]–[27].

The four major subfamilies of ants (i.e., the Ponerinae, Myrmicinae, Formicinae and Dolichoderinae) are characterized by their diversity, abundance and geographically widespread distribution. It is likely that they diversified at the same time [28]. The Ponerinae and Myrmicinae are thought to dominate the ground and leaf-litter of tropical forests, while the Formicinae and Dolichoderinae came to dominate the arboreal strata [5], [28]. Nevertheless, in the monophyletic subfamily Ponerinae [6], [28]–[30], most of the species from the tribe Platythyreini are arboreal [28], [29], while arboreal species in the other tribe, the Ponerini, are infrequent and belong mostly to the genera *Odontomachus* and *Pachycondyla* (subgenus *Neoponera*) [5], [28]. *Pachycondyla (Neoponera)goeldii* workers, for example, adhere to the plant substrate by means of very powerful pretarsal adhesive pads and their claws [31].

Several traits generally considered primitive are widespread across the phylogeny of the Ponerinae, supporting the hypothesis that these are the plesiomorphic states within this subfamily [5], [28], [32]. This is also true for morphological traits (i.e., there is little morphological difference between workers and queens, the worker caste is monomorphic), social behavior (i.e., there is relatively limited chemical communication between nestmates, and an absence of true trophallactic exchanges) and ecological traits (i.e., small colony sizes, solitary foraging, generalized prey preferences, and simple nest construction) [5], [28], [32]. Nevertheless, the Ponerinae diversified into a great wealth of forms, social organizations and lifestyles, so that they flourished throughout the world [5], [28], [32].

**Figure 1:**
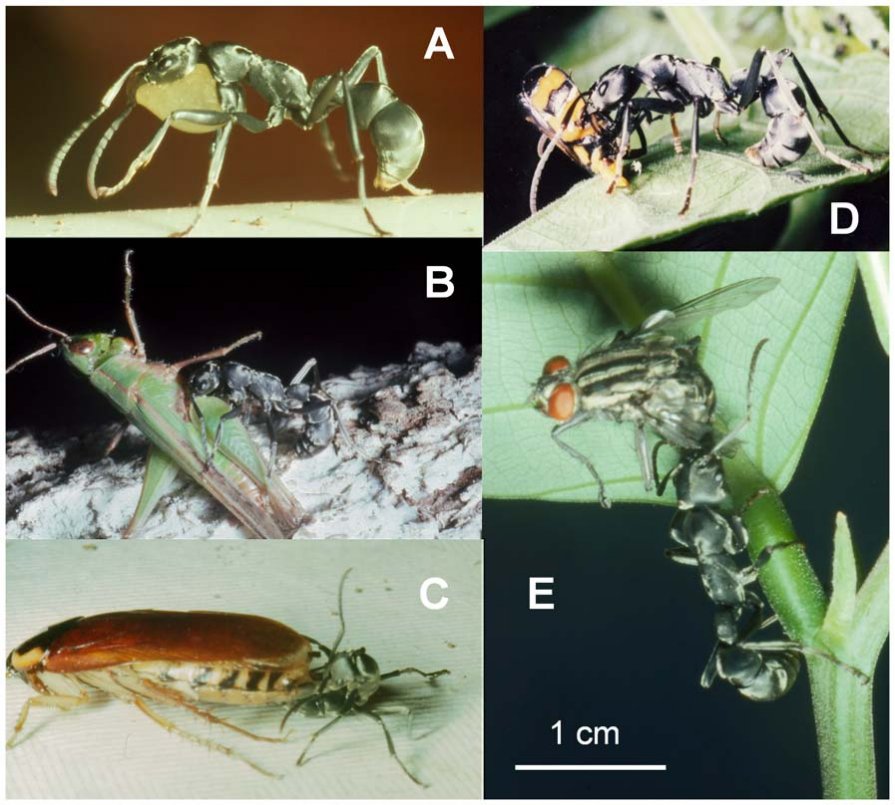
Foraging behavior of *Platythyrea conradti* workers. **A.** A worker transporting a drop of honey under its head and thorax. **B.** Capture of a 3.7-cm-long locust; the worker began to bend its gaster in order to sting. **C.** Capture of a 4.8-cm-long cockroach; the worker, its gaster bent between its legs is stinging the prey on its ventral surface. **D.** A worker transporting a just-captured Tettigometridar (Hemiptera). **E.** A worker having just seized a 1.3-cm-long fly that had settled under a leaf.

This study focuses on *Platythyrea conradti*, an arboreal ponerine ant from the tribe Platythyreini that generally nests between the bark of its host trees and epiphytic ferns [33]. In most of the *Platythyrea* species studied, reproduction is devoted to gamergates (mated workers), while other species also produce winged queens. Exceptionally, *P. conradti* colonies have ergatoid queens that aggressively interact with workers in a dominance hierarchy; high-ranking workers do not reproduce unless the queen dies [34]. The colonies, that can reach ca. 500 workers, frequently shelter commensalist dacetine ants [34], [35]. *Platythyrea conradti* workers compensate for not being able to engage in trophallactic exchanges by transporting large amounts of sugary substances under their heads and thoraxes (Fig. 1A). These sugary substances adhere thanks to surface tension strengths as is known for the workers of some other poneromorph species that use their mandibles in this way, and so can transport only limited loads [36].

I aimed to understand how workers of this non-dominant species can successfully provision their colony by studying (1) their rhythm of activity, (2) their predatory activity, and (3) their reaction *vis-à-vis* competing ant species.

## Materials and Methods

### Study site and experimental procedures

This study was conducted in Yaoundé, Cameroon, both in the field and in the laboratory, on eight colonies of *P. conradti*. All of these colonies were associated with epiphytic ferns of the genus *Platycerium*. For the studies conducted in the laboratory, I gathered three colonies in the field by sawing off segments of branches bearing ferns, and carried the branches back to the laboratory. There, I attached iron rods (60 cm in length) to the middle of the branches, and then fastened the rods to wooden supports that were placed on a table. The workers were free to forage on the tables onto which potted, EFN-producing plants were also set. They deposited visible landmarks (likely corresponding in part to discharged feces; see examples in [32]) while foraging on the table and potted plants where they gathered EFN. I also furnished *ad libitum* small, numbed grasshoppers deposited each night in dishes placed on the table.

### Daily activity rhythm

**Figure 2:**
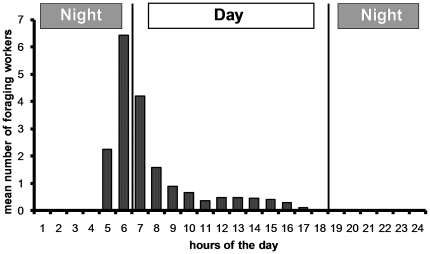
Daily rhythm of activity established from three colonies during the rainy season by counting the number of workers entering and the number of workers leaving their nests during 10 minutes each hour during 17 to 58 series of observations (the means are shown). Between 5:00 and 8:00, most of the foraging activity is related to hunting, while sugary substances are exploited between 5:00 and 17:00.

The rhythm of activity of three colonies was studied in the field during the rainy season. I counted the number of workers entering and the number of workers leaving their nests during 10 minutes each hour during several series of observations spread over 25 days. I conducted 17 to 58 replicates for each hour of the Nyctemeron (out of the 75 possible) and present the means in Fig. 2.

### Captured prey

During the survey on the daily rhythm of activity and during 15 other surveys conducted on five more colonies, I noted what insects were retrieved to the nests by hunting workers.

### Prey-catching behavior

Back in the laboratory, I studied the ants' predatory behavior (between 5:00 and 8:00 a.m.), by conducting a series of tests using termite workers and soldiers (*Macrotermes bellicosus*) and tettigonid larvae of two size classes as prey. The day before each series of tests, I did not provide the colonies with any prey. The day of the tests, I placed prey one by one on the tables serving as the hunting area. The behavioral sequences were recorded through direct observation. Two successive observational periods were separated by at least 30 minutes. A full repertoire of behavioral sequences was first established during preliminary experiments. Referring to this complete list, I recorded each behavioral act performed and the parts of the prey body seized and those stung by the ants. I then built a flow diagram where the transition frequencies between behavioral acts were calculated based on the overall number of transitions between each individual behavioral act (see [18], [19], [21], [23]).

### Reaction to termites and alien ants

I permitted the workers from different ant species (i.e., *Camponotus brutus*, *Crematogaster striatula* and *Oecophylla longinoda*) to forage on the table where the three *P. conradti* colonies had been installed for at least 2 weeks (the workers had deposited visible landmarks). I noted the reactions of the opponents during encounters around drops of honey deposited on the substrate between 10:00 and 11:30 a.m. (10 cases per alien ant species involving 13 to 35 recruited workers; a total of 30 cases).

I also deposited pieces of *Macrotermes* sp. termitaries onto the territories of the *P. conradti* bred in the laboratory (n = 12). I noted the reactions of the foraging workers when encountering the termite workers and soldiers guarding the entrances of the pieces of termitaries and their surroundings.

## Results

### Daily activity rhythm

*Platythyrea conradti* workers are mostly active around dawn, between 5:00 and 8:00. This period corresponds mostly to activities centered on hunting, whereas sugary substances are exploited until 17:00 by only a few workers (Fig. 2). Some workers also simply explore the territory, returning ‘empty-handed’.

### Captured prey

Hunting *P. conradti* workers are able to capture a wide range of insects (Table 1). As mentioned above, they are mostly active around dawn while many insects, inactive at that moment of the Nyctemeron, are resting under leaves or on the trees' branches, and so are relatively easy to capture. This is the case for flies (26% of the prey recorded), grasshoppers and locusts that, during the daytime, can escape by jumping or flying away (Fig. 1). Yet, some crickets and cockroaches were also captured although active nocturnally and very agile (Fig. 1C). Note that swarming ants (winged males and queens) and termites were also frequently captured (Table 1).

**Table 1:** Different captured prey, their weight (or mean weight ± SE) and the ratio with the mean weight of a hunting worker (50.23 mg).

Prey	No. of cases	%	Weight in mg	Ratio / 50.23
Dictyoptera				
Cockroaches 12 mm	8	3.2	44.4	8.84
Cockroach ca. 30 mm	1	0.4	106.5	21.20
*Blatta* sp. ca. 40 mm	1	0.4	142.0	28.26
Termite workers	3	1.2	11.0	0.22
Winged termites (4±0.1 mm); 18±0.1 mg	13	5.3	18.0	0.36
Hemiptera				
Tettigometridae (8 mm)	3	1.2	14.0	0.28
Orthoptera				
Grillidae (crickets) ca. 25 mm	3	1.2	444.0	8.84
Tettigonidae (grasshopper) 10 mm; 49.6±6.8 mg	17	6.9	49.6	0.99
Tettigonidae 12 mm; 80.8±2.1 mg	20	8.1	80.8	1.61
Tettigonidae 22 mm; 257.8±34.4 mg	12	4.9	257.8	5.1
Acrididae (Locust) 10 mm	8	3.2	46.2	0.92
Acrididae 24 mm	4	1.6	770.0	15.33
Acrididae 35 mm	1	0.4	1550.0	30.85
Lepidoptera				
Adult Saturnidae (ca. 20 mm); 470±70 mg	3	1.2	470.0	9.37
Caterpillar 15 mm	6	2.4	88.6	1.76
Hymenoptera (ants)				
*Camponotus* spp. queens	9	3.6	38.0	0.76
*Crematogaster* spp. queens	12	4.9	18.0	0.38
*Camponotus* spp. males	13	5.3	20.0	0.40
*Oecophyla longinoda* queens	6	2.4	45.0	0.90
unidentified males	22	8.9	-	
*Dorylus* sp. Males	4	1.6	83.0	1.66
Diptera				
Mucidae (Flies) (ca. 3 mm)	37	15.0	11.0	0.22
Mucidae (Flies) (ca. 6 mm); 16.5±0.5 mg	27	11.0	16.5	0.33
Tipulidae (Flies) (27 mm)	2	0.8	17.0	0.34
Coleoptera				
Chrysomelidae adults (9±0.2 mm); 99±0.3 mg	7	2.8	99.0	1.97
Chrysomelidae larva (ca. 12 mm)	4	1.6	135.0	2.7
	246	100		

### Prey-catching behavior

**Figure 3:**
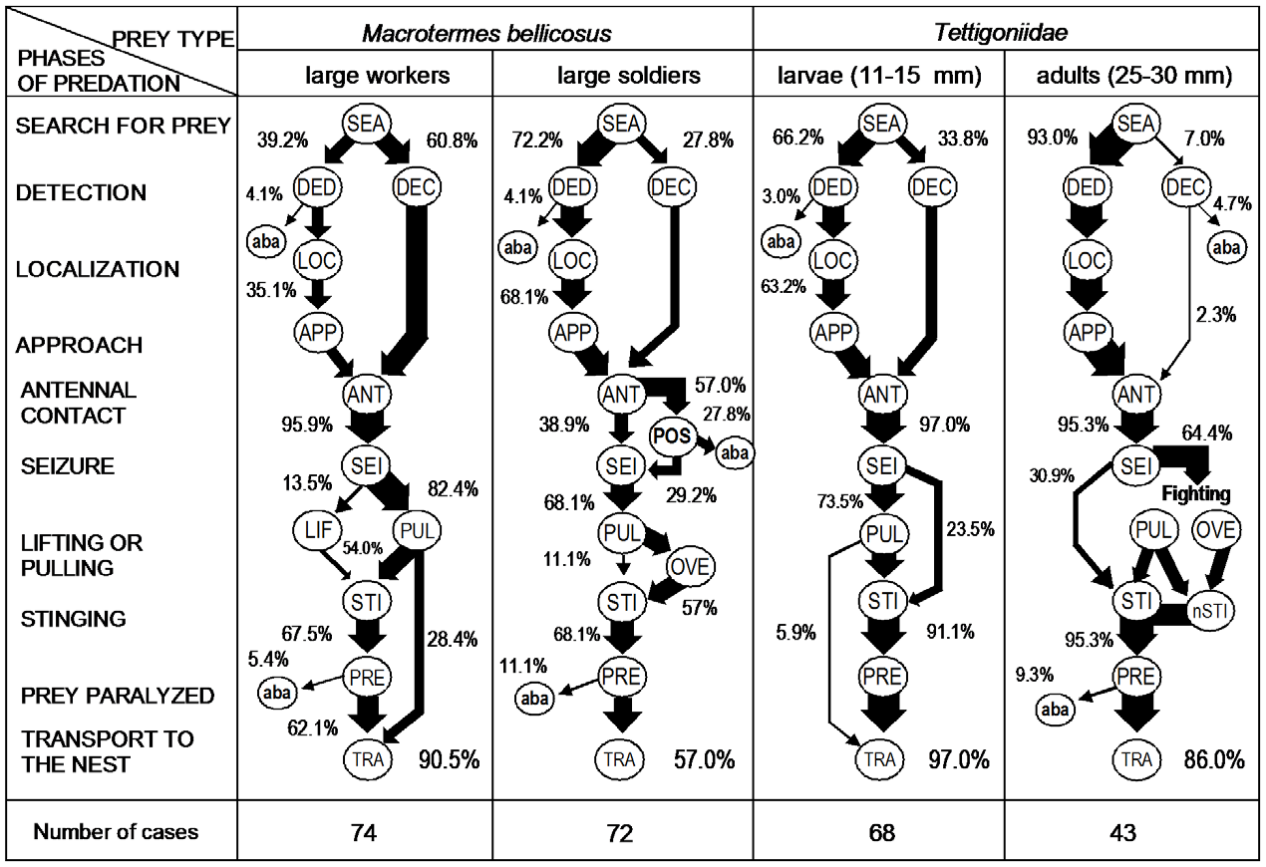
Sequences of predatory behavior in *Platythyrea conradti* workers faced with different prey. The percentages are calculated from the total number of cases. The different phases of predation are shown in the first column. DED, detection at a distance; DEC, detection by contact; POS, crouched posture; OVE, prey overturned; nSTI, numerous, successive stings; aba = prey abandoned.

*Platythyrea conradti* workers can detect prey by contact or from a distance, but only relatively small termite workers were mostly detected by contact (Fig. 3). The larger the prey, the more they were detected from a distance. A brief antennal contact preceded the seizure of the prey body; the prey, whatever its size, was never seized by an appendage, nor by its head. Prey were rather seized by the thorax (termite workers: 71.8%; N = 71; termite soldiers: 69.4%; N = 49; small tettigonids: 74.2%; N = 68; large tettigonids: 56.1%; N = 41) rather than by the abdomen. The differences were not significant (Kruskall-Wallis test: H_3, 8_ = 1.17; P = 0.76). Small prey could be retrieved without being stung (28.4% and 5.9% for termite workers and small tettigonid larva, respectively), while large prey were always stung after being lifted, pulled backward or overturned. We noted that large tettigonids struggled, and were stung numerous times in succession before being definitively mastered. In all cases, the prey were stung on their ventral surface, where the neural chain passes, hastening paralysis. Prey that were abandoned after being stung were later retrieved.

Some termite soldiers immobilized themselves when workers crouched in front of them (“POS” in Fig. 3″; see also below).

Prey were retrieved independently of their size by a single worker (i.e., workers did not recruit nestmates to retrieve large items). During observations conducted both in the field and in the laboratory, we noted that when capturing relatively large prey, such as 3.5-cm-long locusts or 4-cm-long cockroaches (see Table 1), the workers first cut them into two pieces, always retrieved the anterior part, and then returned to the site of capture to retrieve the distal part of the prey. Occasionally, they dragged the large prey (or parts of prey) backward along the vertical zones leading to their nests.

### Reaction to termites and alien ants

**Figure 4:**
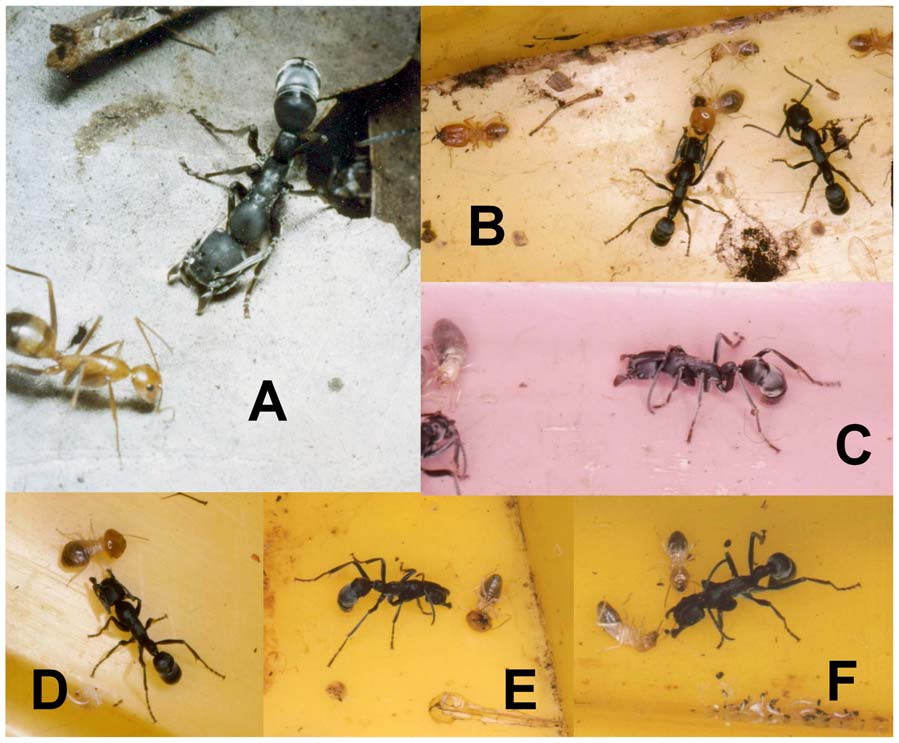
*Platythyrea conradti* workers facing competing ant species or termites. They crouch with their mandibles open, antennae folded backwards. **A.** The competing ant is a *Camponotus brutus* worker. **B–E.** Different *P. conradti* workers are facing *Macrotermes bellicosus* termites. They remain in this posture until the prey, numbed by the probable emission of a chemical substance, roll over, legs batting the air. B. a termite soldier is laying on its back, mandibles crossed. It had previously attacked the crouching *P. conradti* worker, but its mandibles slid due to the hard cuticle of the ant's cephalic capsule. This explains why these ants fold their antenna backward while crouching. C. In certain cases the termite can move, the worker remaining in its crouching posture. D. A crouching *P. conradti* worker, mandibles opened, is approaching a termite. E. It slowly followed the termite until it rolled over. F. Then, another termite arrived.

After a piece of termitary was deposited on the territory of a *P. conradti* colony, the workers faced both termite soldiers and workers that were defending their nests by crouching with their mandibles wide open, and antennae folded backward so that the termite soldiers were not able grab them by an antenna; instead, the soldier's mandibles slipped off of the hard cuticle of the ant's cephalic capsule (see Fig. 4B). Due to their role as guards, the termites did not retreat; rather, they confronted the crouching *P. conradti* workers. After 5 to 15 minutes, these termites began to shake. Then, they fell down, and rolled onto their backs, their legs batting the air (Fig. 4B–E). After ca. 30 minutes, termite nymphs had closed the entrances to the pieces of termitaries with dejections and dirt so that all of the termites left outside were killed by the *P. conradti* workers and retrieved as prey.

This crouching posture was also noted in all cases when the *P. conradti* workers were confronted with alien ant species (see Fig. 4A). These alien ants, even if numerous, little by little abandoned the sugary substance around the zone where the *P. conradti* workers were crouching. While the latter moved forward very slowly, the alien ants progressively retreated so that they ended up abandoning the sugary substances in all 30 cases tested. It is likely that the *P. conradti* workers emit volatile chemical substances that repel alien ants, while they acted as offensive compounds against termites during the previous experiment.

## Discussion

This study demonstrates that *P. conradti* workers, which forage solitary on their host trees, are generalist predators like other arboreal ants, including ponerine species [24], [26], [27]. This permits them to overcome the relative scarcity of prey in the restricted environment constituted by their host tree crown plus tree crowns situated nearby.

The sequence of predation noted here is comparable to the one typically described for generalist ground-nesting ponerine ants; however, like for other arboreal ponerine species, the phase of antennal palpation was reduced to a simple contact immediately followed by the seizure of the prey body, with the gaster already bent, ready to sting. Also, the effect of the venom is immediate compared to that of ground-dwelling species [37]. Even relatively large prey, such as 3-to-4-cm-long cockroaches and locusts, were immediately paralyzed though they were stung successively several times. This rapidity permits arboreal ponerine ants to seize, sting and paralyze insects before they drop, jump or fly away [26]–[27]. Like other Ponerinae [24], *P. conradti* did not sting small prey, illustrating a case of behavioral flexibility.

*Platythyrea conradti* workers are true solitary hunters as, in our experimental conditions, we never noted them recruiting nestmates at short- or long-range, whatever the size of the prey encountered (although they do use scent trails to recruit nestmates at long-range when they find large, sugary food sources; [35]). In the same situation, *P. modesta* workers do recruit nestmates [27] and territorially-dominant arboreal ants and plant-ants hunt in a group, seizing prey by their appendages and spread-eagling them. Spread-eagling generally suffices to numb or kill the prey, and the use of venom has only been noted for some species ([21], [24] and references cited therein).

The success of *P. conradti* workers in capturing a wide range of prey is in large part due to the fact that they hunt near dawn while diurnal insects are in their phase of inactivity, resting on branches or under leaves. Nevertheless, they also easily capture nocturnal insects such as crickets and cockroaches (see Table 1 and Fig. 1). Like other arboreal Ponerinae [26], [27], *P. conradti* also capture termites. In fact, most ponerine species hunt termites [5], [24], [28], [31]; exceptions have been noted for highly specialized species [24].

Another particularity of *P. conradti* foragers is their crouching behavior (mandibles wide open, antennae folded backward) when faced with termites defending their nests that, due to their role as guards, confront these crouching ants and inevitably end up on their back, legs batting the air. Because crouching workers hold their mandibles wide open, one can hypothesize that volatile chemicals are secreted by their mandibular gland whose exit ducts open on the inner surface of the mandible's articulation [31]. There is an urgent need for future studies on *P. conradti* to provide evidence in support of this hypothesis especially as the mandibular glands of certain ponerine ants are known to produce toxic secretions, such as terpenoids [38].

The same behavior was noted against competing ant species that, in this case, retreat further and further away (abandoning the sugary substances in our experiment). Here, the emitted volatiles act as a repellent; whereas, when workers of the plant-ant *Tetraponera penzigi* crouch when encountering alien *Crematogaster* ants sharing the same host tree, the emitted mandibular gland secretions - similar to those of the *Crematogaster* - probably act as chemical camouflage [39].

It is likely that *P. conradti* workers use this crouching behavior to complement visible landmarks that they deposit on branches and leaves (as well as on the tables in laboratory settings) to maintain a kind of territory in the canopy. Due to *P. conradti*'s rhythm of activity, these territories generally overlap with those of territorially-dominant arboreal ants. Yet, in old, abandoned cocoa tree plantations where some trees bear epiphytic *Platycerium*, we noted that workers of territorially-dominant species avoided the territories defended by *P. conradti* colonies. The status of such colonies corresponds to the definition of “sub-dominant species” or species that generally act as non-dominant but are able, under certain conditions – here the presence of *Platycerium* as an adequate nesting site - to defend territories in the same way as do territorially-dominant ants [11], [13], [14]; for instance, *P. laboriosa* and *P. modesta* (cited above) have also been noted acting as sub-dominant species in cocoa tree plantations [40], [41].

In conclusion, *P. conradti* is an arboreal ponerine species whose colonies nest in the forest canopy, and whose workers transport nectar and honeydew using surface tension strengths. Its ability to successfully capture prey is aided by its rhythm of activity as its workers hunt around dusk, when most of their prey, diurnal, are inactive. They nevertheless are also able to capture termites and agile, nocturnal insects. Finally, through the use of landmarks and their crouching posture, foraging workers are able to repel competing ant species, including territorially-dominant arboreal ants. The latter behavior also serves to paralyze termites defending their nests.
